# Predicting All-Cause Mortality Risk in Atrial Fibrillation Patients: A Novel LASSO-Cox Model Generated From a Prospective Dataset

**DOI:** 10.3389/fcvm.2021.730453

**Published:** 2021-10-18

**Authors:** Yu Chen, Shiwan Wu, Jianfeng Ye, Muli Wu, Zhongbo Xiao, Xiaobin Ni, Bin Wang, Chang Chen, Yequn Chen, Xuerui Tan, Ruisheng Liu

**Affiliations:** ^1^Department of Cardiology, The First Affiliated Hospital of Shantou University Medical College, Shantou, China; ^2^Clinical Research Center, The First Affiliated Hospital of Shantou University Medical College, Shantou, China; ^3^Institute of Cardiac Engineering, The First Affiliated Hospital of Shantou University Medical College, Shantou, China; ^4^Department of Molecular Pharmacology and Physiology, Morsani College of Medicine, University of South Florida, Tampa, FL, United States

**Keywords:** atrial fibrillation, machine learning, prediction model, mortality, risk factors

## Abstract

**Background:** Although mortality remains high in patients with atrial fibrillation (AF), there have been limited studies exploring machine learning (ML) models on mortality risk prediction in patients with AF.

**Objectives:** This study sought to develop an ML model that captures important variables in order to predict all-cause mortality in AF patients.

**Methods:** In this single center prospective study, an ML-based mortality prediction model was developed and validated using a dataset of 2,012 patients who experienced AF from November 2018 to February 2020 at the First Affiliated Hospital of Shantou University Medical College. The dataset was randomly divided into a training set (70%, *n* = 1,223) and a validation set (30%, *n* = 552). A total of 122 features were collected for variable selection. Least absolute shrinkage and selection operator (LASSO) and random forest (RF) algorithms were used for variable selection. Ten ML models were developed using variables selected by LASSO or RF. The best model was selected and compared with conventional risk scores. A nomogram and user-friendly online tool were developed to facilitate the mortality predictions and management recommendations.

**Results:** Thirteen features were selected by the LASSO regression algorithm. The LASSO-Cox model achieved an area under the curve (AUC) of 0.842 in the training dataset, and 0.854 in the validation dataset. A nomogram based on eight independent features was developed for the prediction of survival at 30, 180, and 365 days following discharge. Both the time dependent receiver operating characteristic (ROC) and decision curve analysis (DCA) showed better performances of the nomogram compared to the CHA_2_DS_2_-VASc and HAS-BLED models.

**Conclusions:** The LASSO-Cox mortality predictive model shows potential benefits in death risk evaluation for AF patients over the 365-day period following discharge. This novel ML approach may also provide physicians with personalized management recommendations.

## Introduction

AF is one of the most common chronic cardiovascular health problems globally (1–3). In Europe and the USA, 2–3 % of the population suffers from AF (4), and it is estimated that AF will affect 6–12 million people in the USA by 2,050 and 17.9 million people in Europe by 2,060 (5, 6). The incidence of AF is not high among young people but increases with age, reaching more than 10 % in those >80 years of age (7). The inevitable global aging of the population, combined with a cumulative increase in chronic cardiovascular diseases, will lead to considerable growth in the number of AF patients in the next few decades. AF is associated with a nearly five-fold increased risk of ischemic stroke (8, 9), and provokes significant increases in all-cause mortality along with important financial burden (10, 11). Consequently, higher risk of all-cause mortality associated with AF has become a significant public health issue (1, 11–13).

Several classic risk scores, including CHA_2_DS_2_-VASc and HAS-BLED scores, predict clinical outcomes, such as for stroke, bleeding and mortality (14–17). Machine learning can learn to identify the underlying pattern and classes from multidimensional data by utilizing computational algorithms (18). Based on novel ML algorithms, more accurate and intelligent models, such as the Global Anticoagulant Registry in the Field (GARFIELD)-AF risk model and the Multilayer Neural Network artificial intelligence model, have been developed (19–21). In contrast to the high awareness regarding clinical outcomes of AF in Europe and the USA, there is limited knowledge for East Asia. In addition, few ML models have used multi-dimensional features to predict future mortality of AF patients.

Advances in supervised ML allow the recognition and translation of multi-dimensional data into valuable models (21, 22). The use of machine learning for predicting clinical outcomes may enable physicians to improve efficiency, reliability, and accuracy of management decisions. In the present study, we used multiple ML approaches that included LASSO feature selection and the Cox proportional hazards regression model to predict all-cause mortality outcome over the 30–365-day period after discharge in patients with AF.

## Methods

### Study Cohort

For machine learning model construction, a prospective observational study was undertaken using data from patients who were hospitalized for evaluation and treatment of AF between November 2018 and February 2020 at the First Affiliated Hospital of Shantou University Medical College. Inclusion criteria were a diagnosis of AF and availability of complete data concerning clinical indicators for evaluating AF and follow-up. The diagnosis of AF required recording the heart rhythm by electrocardiogram (ECG). Three diagnostic criteria shown by ECG are: (1) absolutely irregular RR intervals, (2) no discernible, distinct P waves, and (3) an episode lasting at least 30 s. Many individuals with AF have both symptomatic and asymptomatic episodes. The exclusion criteria were pregnant women, age ≤ 18, or patients who refused follow-up.

### Data Collection

A systemic clinical evaluation for AF was conducted during the hospitalization when patients were enrolled. Overall, 122 variables were initially used for the selection of key features (Supplementary Table 1), which included medical histories, physical examinations, laboratory examination results, medications, comorbidities, ultrasonic cardiogram, CHA_2_DS_2_-VASc score, and HAS-BLED score. Follow-up by outpatient follow-up and/or telephone interview was carried out at 30, 180, and 365 days after discharge. The main outcome of the AF cohort was all-cause death.

This study complied with the principles of the Declaration of Helsinki and was approved by the Ethics Committee of the First Affiliated Hospital of Shantou University Medical College. All participants provided written informed consent to participate in this study. All procedures were performed in conformity with the European Society of Cardiology guidelines (23).

### Variable Selection and Model Development

Due to the 122 variables present in the dataset, conducting variable selection was necessary and could lead to improved prediction performance. Both the LASSO algorithm (24) and RF (25) were used to select the features for model training. The top 20 predictor variables were chosen using RF based on relative variable importance (26).

We used five algorithms, including Cox regression, RF, support vector machines (SVM) (27), backpropagation neural networks (BP-NN) (28), and gradient boosting (GB) (29), to train models using the variables that were selected by LASSO and RF. Ultimately, 10 models, including LASSO-Cox, LASSO-RF, LASSO-SVM, LASSO-BP-NN, LASSO-GB, RF-Cox, RF-RF, RF-SVM, RF-BP-NN, and RF-GB, were established.

### Statistical Analyses and Model Performance Measures

Statistical analyses were performed using SPSS 23.0 (Inc., Chicago, Illinois, USA), X-tile 3.6.1 (30), and R (version 4.0.2; R Foundation for Statistical Computing, Vienna, Austria) software. Continuous variables are presented as the mean ± standard derivation. We used multiple imputation to account for missing data on continuous variables if missing data was <30% (31). Missing values were imputed using the “mice” package. Categorical variables are presented as numbers and percentages. Statistical differences of continuous variables were examined by two-tailed *t*-tests or Mann-Whitney *U* tests. Categorical variables were analyzed by the chi-square test or Fisher exact test. Various *R* packages were used to conduct this study. The glmnet package was used for logistic regression with LASSO regularization (32). Random forest, e1071, neural net, and gbm packages were used for the RF, SVM, BP-NN, and GB models, respectively (29, 33).

The predictive accuracy of the LASSO-Cox model was compared with the performances of CHA_2_DS_2_-VASc and HAS-BLED scores. The performances of the models were assessed by the AUC derived from receiver operating characteristics curves. A nomogram for predicting the 30-, 180- or 360-day survival was established using the LASSO-Cox regression model, and the cut-off value for mortality risk stratification was calculated. The nomogram and calibration plots were generated with the rms package. The pROC package was used to plot ROC curves. Kaplan-Meier curves were produced using the survival package. *P* < 0.05 was considered to indicate statistical significance.

## Results

### Patient Baseline Demographics

**Figure 1:**
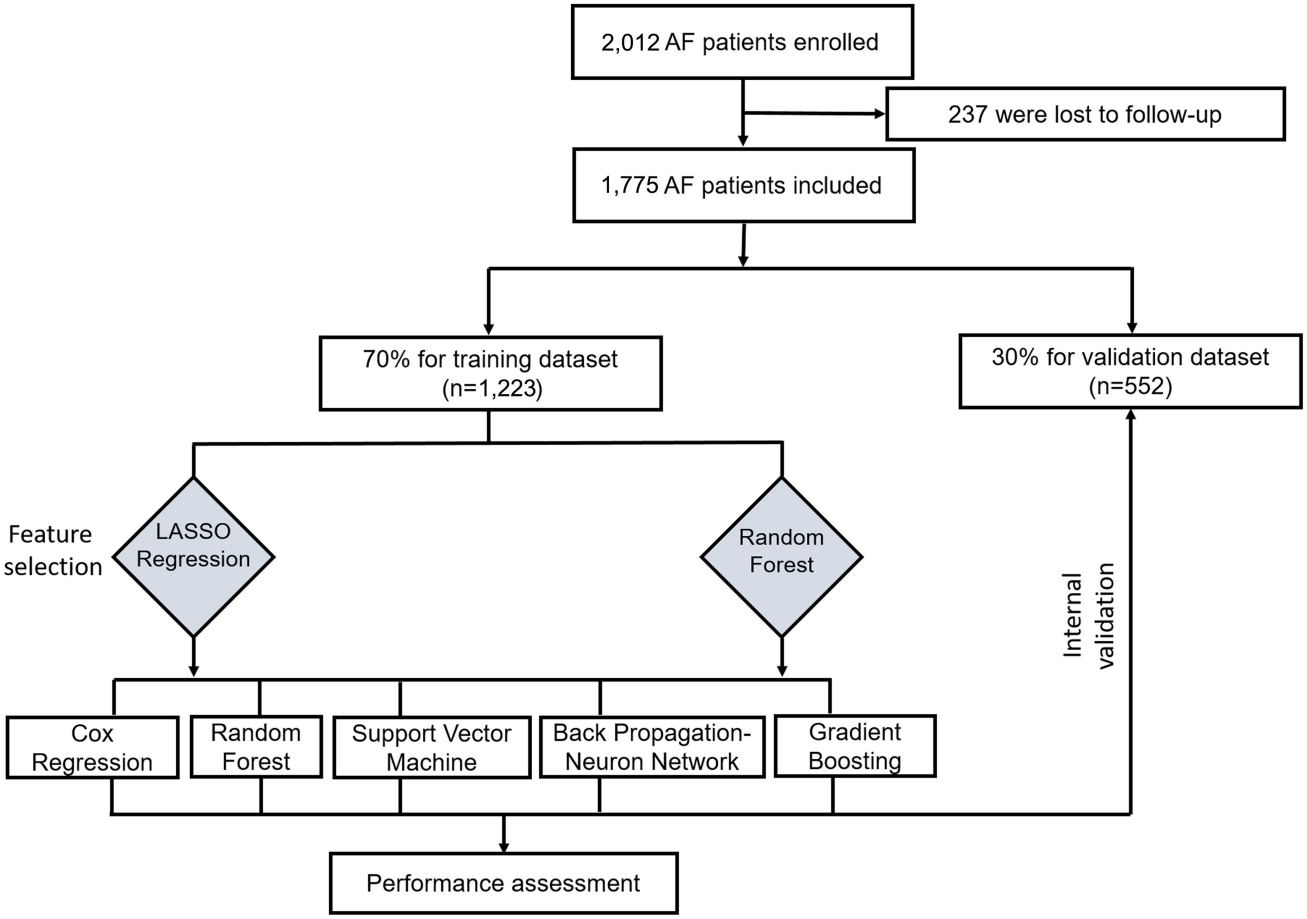
Flow chart for the training and valuation of models. LASSO, least absolute shrinkage and selection operator.

This study was conducted according to the flow chart shown in Figure 1. Eligible study participants consisted of 1,775 AF patients. A total of 1,223 AF patients were randomly assigned in the training dataset and 552 patients in the validation dataset. Baseline characteristics of the study cohort are shown in Table 1. The mean age was 69.22 years (SD = 12.05 years) for the training dataset and 69.02 years (SD =11.65 years) for the validation dataset. The mean CHA_2_DS_2_-VASc was 3.37 (SD = 1.18) in the training set and 3.19 (SD = 1.80) in the validation set. There were no significant differences in diabetes, atherosclerosis, prior stroke, heart failure, cerebral hemorrhage, cancer, renal insufficiency, bleeding, current smoker status, statin medication, and urine ketone bodies in the training set compared with the validation set. An all-cause mortality end point event occurred for 194 of the 1,775 patients (10.9%, 111 males and 83 females), 143 in the training set (11.7%) and 51 in the validation set (9.2%). There was no significant difference in all-cause death rate between the training and validation set.

**Table 1:** Baseline characteristics of the AF prospective cohort.

**Variable**	**Training set (*n* = 1,223)**	**Validation set (*n* = 552)**	***P*-value**
Age (years)	69.22 ± 12.05	69.02 ± 11.65	0.749
Gender: male	682 (55.8)	334 (60.5)	0.062
Diabetes mellitus	324 (26.5)	163 (29.5)	0.184
Atherosclerosis	446 (36.5)	202 (36.6)	0.959
Hypertension	759 (62.1)	297 (53.8)	<0.001
Prior stroke	319 (26.1)	127 (23.0)	0.167
Cerebral hemorrhage	36 (2.9)	9 (1.6)	0.103
Heart failure	319 (26.1)	119 (21.6)	0.041
Cancer	80 (6.5)	30 (5.4)	0.371
Renal insufficiency	154 (12.6)	67 (12.1)	0.788
Bleeding	47 (3.8)	22 (4.0)	0.886
Current smoker status	347 (28.4)	150 (27.2)	0.603
Alcohol	87 (7.1)	31 (5.6)	0.241
Anticoagulant treatment	696 (56.9)	299 (54.2)	0.281
β-Blocker treatment	546 (44.6)	237 (42.9)	0.502
Digoxin treatment	398 (32.5)	192 (34.8)	0.354
Statin medication	486 (39.7)	230 (41.7)	0.443
WBC (10^9^/L)	8.43 ± 3.71	8.30 ± 3.29	0.47
KET			0.463
0	1,093 (89.4)	497 (90.0)	
1	124 (10.1)	52 (9.4)	
2	6 (0.5)	2 (0.4)	
3	0 (0.0)	1 (0.2)	
Lymphocyte ratio	20.20 ± 10.65	20.59 ± 10.93	0.48
Creatinine (mmol/L)	119.05 ± 78.09	117.84 ± 73.22	0.758
RDW-CV (%)	14.53 ± 2.01	14.36 ± 1.79	0.962
Platelet (10^9^/L)	205.18± 69.61	202.30 ± 73.27	0.426
Platelet distribution width (%)	15.62 ± 1.98	15.67 ± 1.93	0.648
GLU (mmol/L)	7.00 ± 3.65	6.76 ± 3.28	0.191
BUN (mmol/L)	7.77 ± 4.75	7.92 ± 5.17	0.542
CHE (U/L)	6.42 ± 1.96	6.41 ± 1.96	0.927
MAO (U/L)	4.89 ± 2.35	4.91 ± 2.68	0.862
Neutrophils/lymphocytes (%)	5.85 ± 6.61	5.96 ± 7.19	0.748
CHA_2_DS_2_-VASc score			0.51
0	59 (4.8)	33 (6.0)	
1	139 (11.4)	76 (13.8)	
2	198 (16.2)	99 (17.9)	
3	256 (20.9)	103 (18.7)	
4	250 (20.4)	110 (19.9)	
5	169 (13.8)	68 (12.3)	
6	100 (8.2)	46 (8.3)	
7	42 (3.4)	12 (2.2)	
8	10 (0.8)	5 (0.9)	

*Data are represented as mean ± standard deviation or frequency (percentage). WBC, White blood cell; KET, Urine ketone body; RDW-CV, Red cell volume distribution width - coefficient of variation; GLU, Blood glucose; BUN, Blood urea nitrogen; CHE, Cholinesterase; MAO, Monoamine oxidase*.

### Feature Selection and Model Performance Comparison

**Figure 2:**
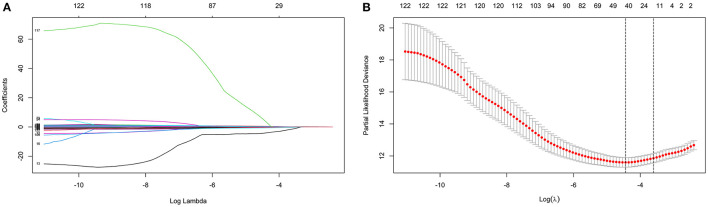
Identification of variables using the least absolute shrinkage and selection operator (LASSO) regression algorithm. The numbers above the graph represent the number of variables involved in the LASSO model. **(A)** LASSO coefficient profiles of the 122 variables. **(B)** Identification of the optimal penalization coefficient λ in the LASSO model. The partial likelihood deviance is plotted against log (λ), where λ is the tuning parameter. Red dots indicate average deviance values for each model with a given λ, and partial likelihood deviance values are shown, with error bars representing s.e. The dotted vertical lines are plotted at the value selected using the 10-fold cross-validation and 1 – s.e. criteria.

**Figure 3:**
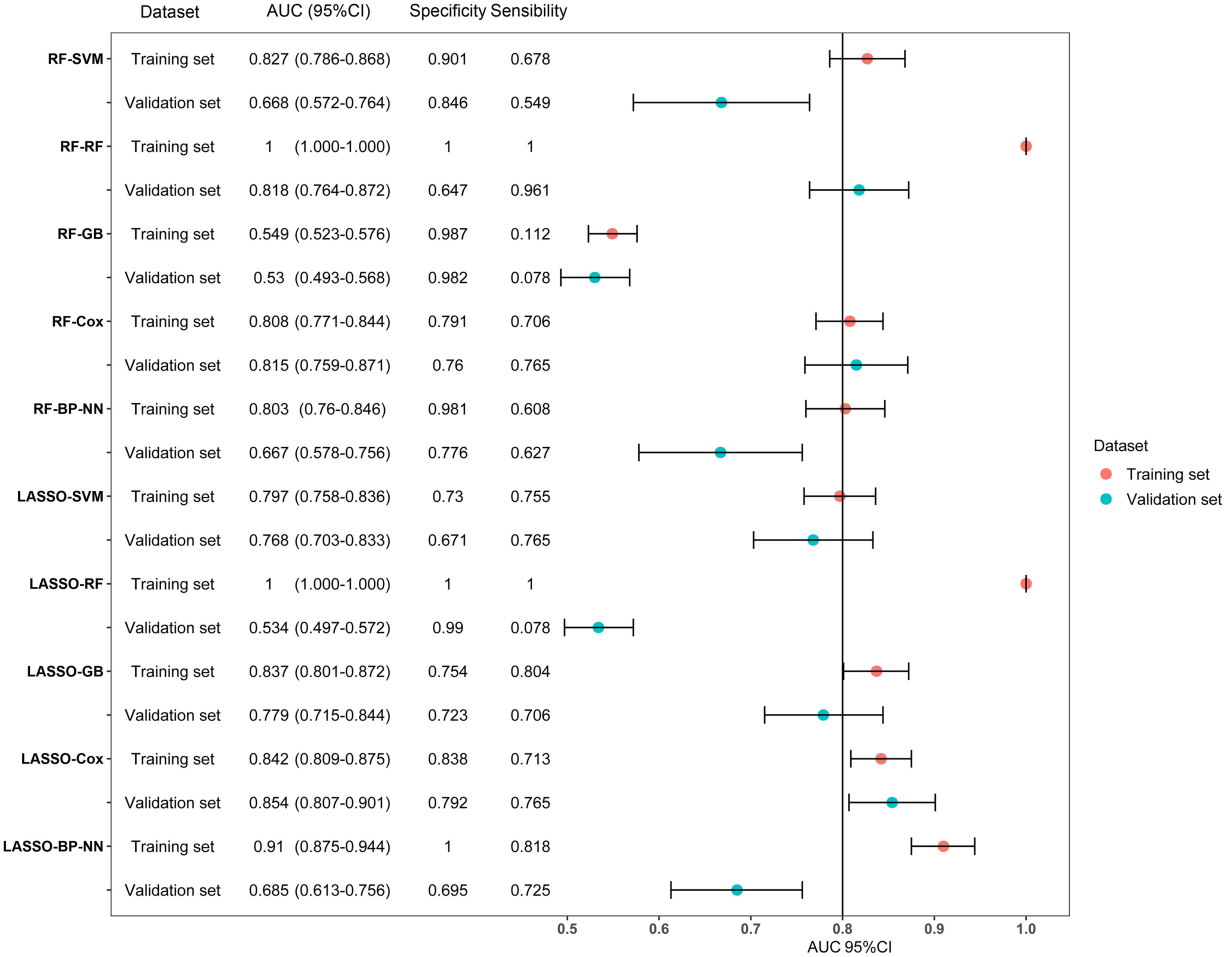
Forest plot of area under the curves (AUC) of the training and validation datasets for the ten models. AUCs are shown with 95 percent confidence intervals for training set and validation set in each algorithm group. RF, random forest; SVM, support vector machine; GB, gradient boosting; BP-NN, backpropagation neural network; LASSO, least absolute shrinkage and selection operator.

LASSO coefficient profiles of the 122 variables and ten-fold cross-validation for tuning parameter selection in the LASSO model are shown in Figure 2. Thirteen variables were selected by the LASSO regression algorithm, including CHA_2_DS_2_-VASc, stroke, cancer, red cell volume distribution width-coefficient of variation (RDW-CV), statin medication use, lymphocyte ratio, neutrophil-to-lymphocyte ratio, basophilic granulocyte number, urine ketone body (KET), blood glucose (GLU), blood urea nitrogen (BUN), cholinesterase (CHE), and monoamine oxidase (MAO). In addition, the top-20 variables were selected by the RF algorithm (Supplementary Table 2). Next, we built 10 models using these two sets of selected features, and their prediction performances were described using AUC, sensitivity, and specificity (Figure 3). The key performance of machine learning was evaluated by AUC.

Among the 10 models, LASSO-BP-NN had the highest AUC (0.910, 95% CI: 0.875–0.944) in the training dataset, but a relatively low AUC (0.685, 95% CI: 0.613–0.756) in the validation dataset. The LASSO-Cox model, over the 1-year follow-up, achieved an AUC of 0.842 (95% CI: 0.809–0.875) in the training dataset, and an AUC of 0.854 (95% CI: 0.807–0.901) in the validation dataset. Due to the very good performances in both the training set and validation set, the LASSO-Cox regression was chosen as the best model.

### Nomogram Construction

Based on the Cox proportional hazards regression analysis, we identified eight independent risk factors in the training cohort. CHA_2_DS_2_-VASc (hazard ratio, HR = 1.188, *P* = 0.002), stroke (HR = 1.717, *P* = 0.008), cancer (HR = 2.208, *P* = 0.002), statin medication use (HR = 0.341, *P* < 0.001), KET (HR = 1.730, *P* = 0.006), BUN (HR = 1.037, *P* = 0.003), CHE (HR = 0.889, *P* = 0.032), and MAO (HR = 1.133, *P* < 0.001) were all significantly associated with mortality in AF patients (Supplementary Table 3).

**Figure 4:**
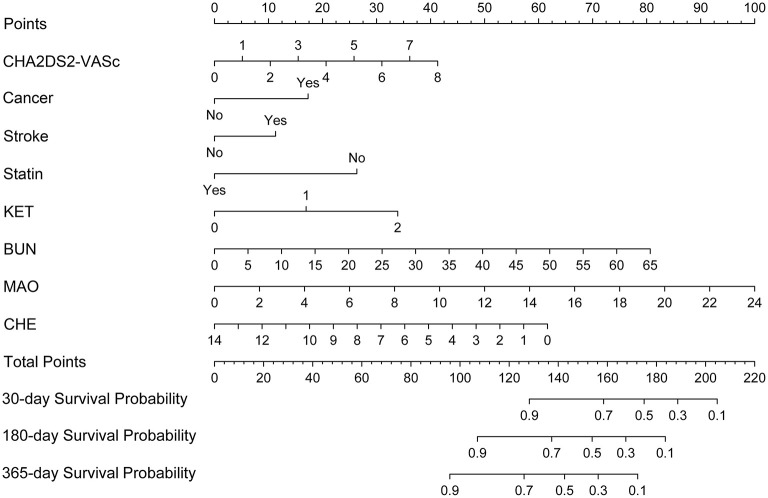
Nomogram for predicting 30-, 180-, and 365-day survival probabilities for AF patients. To calculate patient survival probabilities, obtain points for each covariate value by dropping a vertical line from the points axis to the value of each covariate, calculate the total points obtained from all eight covariate values, and then drop a vertical line from the total points axis to locate the associated 30-, 180-, or 365-day survival probability. KET, urine ketone body; BUN, blood urea nitrogen; CHE, cholinesterase; MAO, monoamine oxidase.

A nomogram based on the eight independent features from the training cohort was developed for the prediction of the 30-, 180-, and 365-day survival (Figure 4). The nomogram demonstrated that MAO contributes the most to survival, followed by CHE, KET, BUN, CHA_2_DS_2_-VASc, stroke, statin use, and cancer. The total score, obtained by adding the scores for each of the eight features, helped in estimating the 30-, 180-, and 365-day survival rate for each individual patient.

### Validation and Calibration of the Nomogram

ROC curves were used to evaluate the predictive ability for 30-, 180-, and 365-day survival in both the training and validation sets. Our Cox model demonstrated good discriminative ability in both the training (30-day AUC: 0.848, 180-day AUC: 0.826, 365-day AUC: 0.762) and validation (30-day AUC: 0.834, 180-day AUC: 0.788, 365-day AUC: 0.841) datasets for the 30-, 180-, and 365-day survival rates (Supplementary Figure 1). The calibration plots of our nomogram also showed optimal agreement between the actual observations and the predicted outcomes both in the training set and validation set (Supplementary Figure 2) for all time points. Thus, the above nomogram-based results displayed good accuracy for predicting the 30-, 180-, and 365-day survival of AF patients.

### Comparison of the Nomogram With CHA_2_DS_2_-VASc and HAS-BLED Models for Predictive Performance

**Figure 5:**
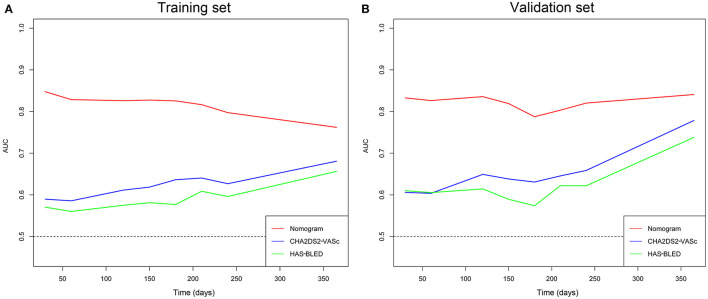
Time-dependent ROC of the nomogram compared with CHA_2_DS_2_-VASc and HAS-BLED models in the training and validation sets. **(A)** Training set. **(B)** Validation set.

**Figure 6:**
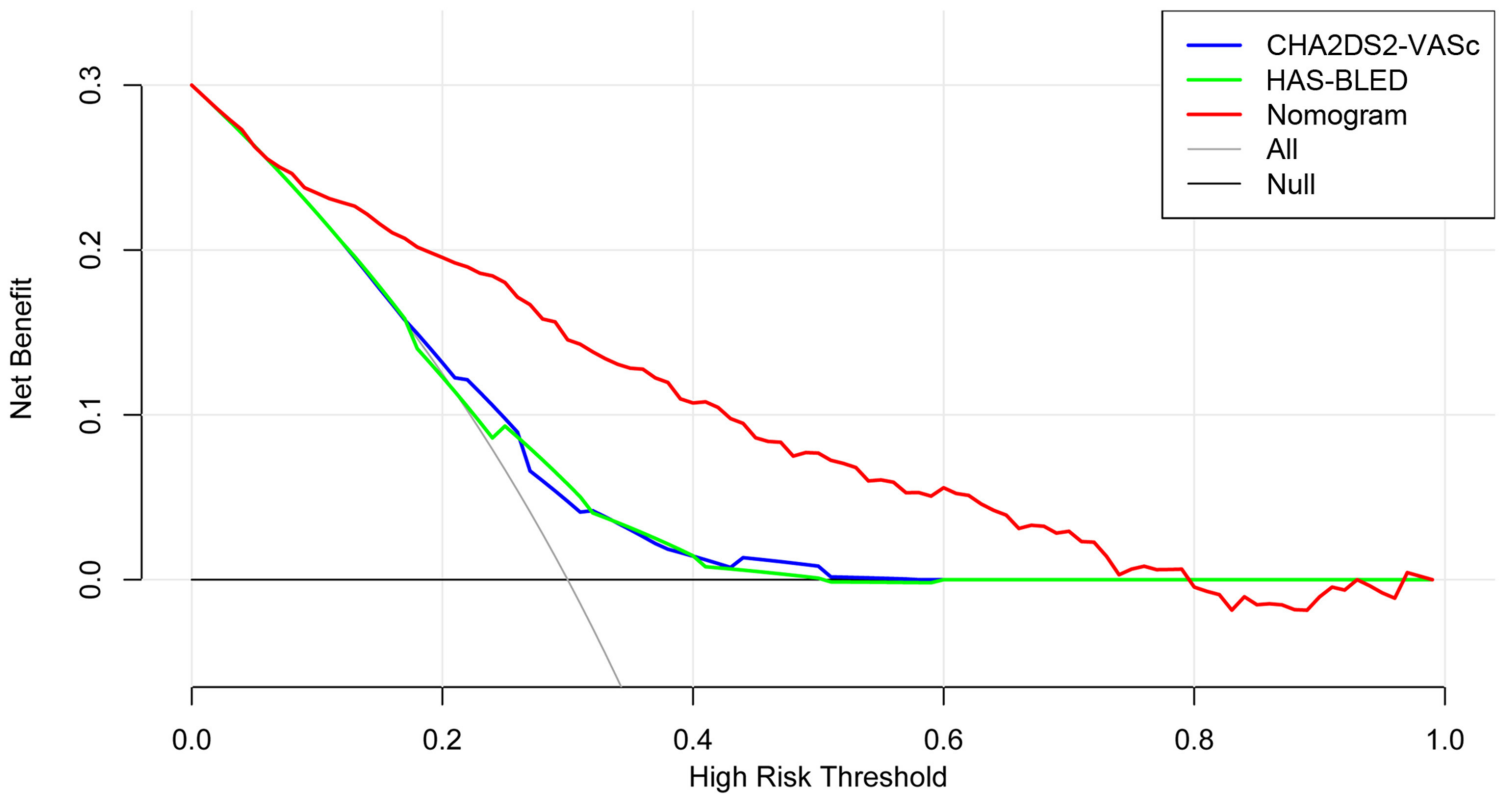
Decision curve analysis of the nomogram, CHA_2_DS_2_-VASc score and HAS-BLED score. The y-axis represents the net benefit and the x-axis represents the threshold probability. The null plot represents the assumption that no patients survive, while the all plot represents the assumption that all patients survive at a specific threshold probability.

The time-dependent ROCs of the training and validation sets (Figure 5) based on the nomogram were higher than those based on the traditional CHA_2_DS_2_-VASc and HAS-BLED models. These results indicate that our nomogram has greater potential for accurately predicting prognosis compared to the traditional models. DCA was performed to compare the net benefit of the nomogram with that of the traditional CHA_2_DS_2_-VASc and HAS-BLED scores. Compared to the CHA_2_DS_2_-VASc and HAS-BLED scores, the curve of our nomogram showed larger net benefit (Figure 6). We further converted the nomogram to a web calculator for the clinician's convenience (https://afnom.shinyapps.io/DynNomapp/).

In addition, the optimal cut-off point was determined using the X-tile program to accomplish risk stratification. As shown in Supplementary Figure 3, the optimal cut-off point was 0.8. Thus, we stratified the AF patients into a low-risk group (≤0.8) and high-risk group (>0.8). Kaplan–Meier curves showed that the high-risk group exhibited poorer survival than the low-risk group in both the training and validation sets (Supplementary Figure 4).

## Discussion

This study investigated a novel LASSO-Cox model for the prediction of all-cause mortality in patients with AF to identify AF patients at high risk and to provide personalized treatment using a data-driven approach. Several important findings were identified. First, eight independent risk factors predicted all-cause mortality, including CHA_2_DS_2_-VASc score, CHE, KET, BUN, MAO, stroke, statin medication use, and cancer. Second, a LASSO-Cox model for 30-, 180-, and 365-day risk prediction was established and validated. Third, the use of the nomogram and risk stratification enables the prediction of mortality for AF patients.

Machine learning can identify non-linear associations and identify interactions in complex and multidimensional variables. The use of the LASSO ML algorithm for variable selection is a well-established method that has been previously utilized for cancer, heart failure, and AF populations (34–36). The advantages of the LASSO algorithm are high accuracy and stability. Cox proportional hazards regression is a traditional model, that is mainly used to analyze the prognosis of cancer and other chronic diseases. Indeed, our LASSO-Cox model was robust and displayed good discriminatory power in predicting all-cause mortality both in the training and the validation dataset.

There is growing evidence that AF significantly worsens the mortality rate (37–39). Furthermore, AF is an independent risk factor for higher risk of mortality (11). While worse outcomes among AF patients have been confirmed in various studies from Europe and North America, data from East Asia is limited.

Traditional guidelines in AF have focused on identifying patients with different risks of stroke and major bleeding. Several studies have developed and examined prediction models or risk scores in AF patients for stroke, major bleeding, or composite outcomes, although not exclusively for death outcomes (19, 23, 40). Recently, a death risk score based on age, biomarkers, and clinical history (ABC) was developed and performed well in two large independent clinical trial cohorts (41). However, the detection of novel biomarkers such as GDF-15 are not easily performed in developing countries and regions.

In this LASSO-Cox model, not taking statins is an independent risk factor for AF-associated death. As recently reported, the levels of total cholesterol (TC) are non-linearly associated with all-cause mortality, as well as cancer and cardiovascular disease mortality, in the American population (42). Thus, it is necessary to maintain TC in a moderate range by statin medication. The GARFIELD-AF and ROCKET AF studies have shown that heart failure and sudden cardiac death are the major reasons for death of AF patients taking oral anticoagulant medication (38, 43). Death risk prediction in these patients may give rise to more intense management of risk factors, such as valvular heart disease, myocardial dysfunction, and coronary heart disease.

Among the independent risk factors of death, the four common laboratory examination indicators, including MAO, BUN, CHE, and KET, are strongly associated with mortality. Contemporary AF trials show that cardiac-related deaths account for the vast majority of all deaths, whereas stroke and bleeding represent a small fraction (44). In our study, MAO is recognized as the most important mortality risk factor in AF patients. Elevated MAO is known to be associated with liver cirrhosis and chronic congestive heart failure. Recent studies show that MAO is a major source of deleterious reactive oxygen species (ROS), regulating cardiomyocyte aging or death (45, 46). Myocardial ROS are involved in the pathophysiology of cardiovascular diseases such as hypertension and heart failure (47, 48), and are important markers of atrial fibrillation in patients after cardiac surgery (49). Thus, MAO inhibition therapy is protective in several settings of cardiac stresses such as pressure overload heart failure, diabetic cardiomyopathy and chronic ischemic heart disease (47). Further studies exploring the potential relationship between AF and ROS are needed.

Increased BUN levels are mainly triggered by impaired renal function, which might be highly related to the occurrence of ischemic stroke in AF patients despite adequate therapeutic warfarin anticoagulation (50). A Swedish study showed that neoplastic disease and renal failure contribute to the increased risk of all-cause mortality in AF patients, which is consistent with our result (11). Declination of cholinesterase is associated with the advanced liver cirrhosis, hepatic failure, and myocardial infarction. Inhibition of CHE has been reported to directly affect the intrinsic cardiac nervous system (51). In addition, increased levels of KET reflects the severity of diabetes, and AF patients with diabetes mellitus have a higher mortality rate (52–54). Collectively, the above risk factors suggest a renewed emphasis on the management of comorbidities such as liver cirrhosis, renal dysfunction, heart failure, and diabetes mellitus, is essential to improve the overall survival and quality of life in AF patients.

The nomogram could provide clinicians with the opportunity to assess risk of all-cause mortality by using a data-driven approach. An additional strength of the LASSO-Cox model is that the eight predictive factors in this nomogram are widely and easily available internationally. In order to facilitate medical use, the clinical implementation of the LASSO-Cox model can either be based on the nomogram, or preferably an online tool.

### Limitations

Several limitations of this LASSO-Cox model should be considered. First, validation of this model was performed using a dataset generated from a single center. The performance of our LASSO-Cox model in external datasets needs be tested by data from other institutions. Second, the LASSO-Cox model did not include information about biomarkers, such as NT-proBNP and hs-cTnT. However, considering that these biomarkers often require additional examination, thus increasing the difficulty of acquisition, our model has good accuracy and ease of application. Third, multiple imputation for the missing values is a potential source of bias. Nevertheless, multiple-imputation is a commonly used rigorous technique for imputation (55).

## Conclusion

A new LASSO-Cox model for predicting risk of all-cause mortality in patients with AF was successfully developed, and internally validated. The LASSO-Cox model using CHA_2_DS_2_-VASc score, statin medication, medical history (stroke, cancer), and four clinical examination parameters (KET, BUN, MAO, and CHE), performed well and may assist physicians in decision-making when treating AF patients.

## Data Availability Statement

The original contributions presented in the study are included in the article/Supplementary Material, further inquiries can be directed to the corresponding authors.

## Ethics Statement

This study complied with the principles of the Declaration of Helsinki and was approved by the Ethics Committee of the First Affiliated Hospital of Shantou University Medical College. All participants provided written informed consent to participate in this study. All procedures were performed in conformity with European society of cardiology guidelines. All procedures followed were in accordance with the ethical standards of the responsible committee on human experimentation (institutional and national) and with the Helsinki declaration of 1975, as revised in 2000. Informed consent was obtained from all patients for being included in the study.

## Author Contributions

SW, YQC, and XT: concept and design, data analysis and interpretation, critical revision of article, and approval. YC, MW, ZX, XN, and BW: statistics, data analysis, and drafting of article. YC, SW, JY, CC, YQC, and RL: data collection, data analysis, critical revision of article, and approval. All authors read and approved the final manuscript.

## Funding

This work was supported by projects from Grant for Key Disciplinary Project of Clinical Medicine under the High-level University Development Program (2020), Innovation Team Project of Guangdong Universities (2019KCXTD003), Li Ka Shing Foundation Cross-Disciplinary Research Grant (2020LKSFG19B), Funding for Guangdong Medical Leading Talent (2019-2022), and National Natural Science Foundation of China (82073659).

## Conflict of Interest

The authors declare that the research was conducted in the absence of any commercial or financial relationships that could be construed as a potential conflict of interest.

## Publisher's Note

All claims expressed in this article are solely those of the authors and do not necessarily represent those of their affiliated organizations, or those of the publisher, the editors and the reviewers. Any product that may be evaluated in this article, or claim that may be made by its manufacturer, is not guaranteed or endorsed by the publisher.

## Supplementary Material

Supplementary Figure 1 Receiver operating characteristic (ROC) curves for 30-, 180-, and 365-day survival rates. **(A)** Training set. **(B)** Validation set.

Supplementary Figure 2 Calibration curves for the nomogram in the training and validation sets. Y-axis represents the actual survival rate, while the X-axis represents the nomogram-predicted survival rate. The blue dotted line indicates perfect prediction by an ideal model. **(A–C)** 30-, 180-, and 365-day survival rates in the training set. **(D–F)** 30-, 180-, and 365-day survival rates in the validation set.

Supplementary Figure 3 Determination of the cut-off score, for the mortality risk stratification, using the X-tile program. A cut-off score ≤ 0.8 indicates low-risk, and > 0.8 indicates high-risk.

Supplementary Figure 4 Kaplan-Meier curves for the high-risk group and low-risk group in the training and validation sets. **(A)** Training set. **(B)** Validation set.

Supplementary Table 1 The 122 variables and the missing rates collected in the dataset.

Supplementary Table 2 Top-20 variables selected by the RF algorithm.

Supplementary Table 3 Hazard ratios and 95% confidence intervals of 8 variables in the Cox proportional hazards model.
