# Supplementary figures and images for: Predicting All-Cause Mortality Risk in Atrial Fibrillation Patients: A Novel LASSO-Cox Model Generated From a Prospective Dataset
Source: Front Cardiovasc Med. 2021 Oct 18;8:730453. doi: 10.3389/fcvm.2021.730453 (PMC8558306; doi:10.3389/fcvm.2021.730453)

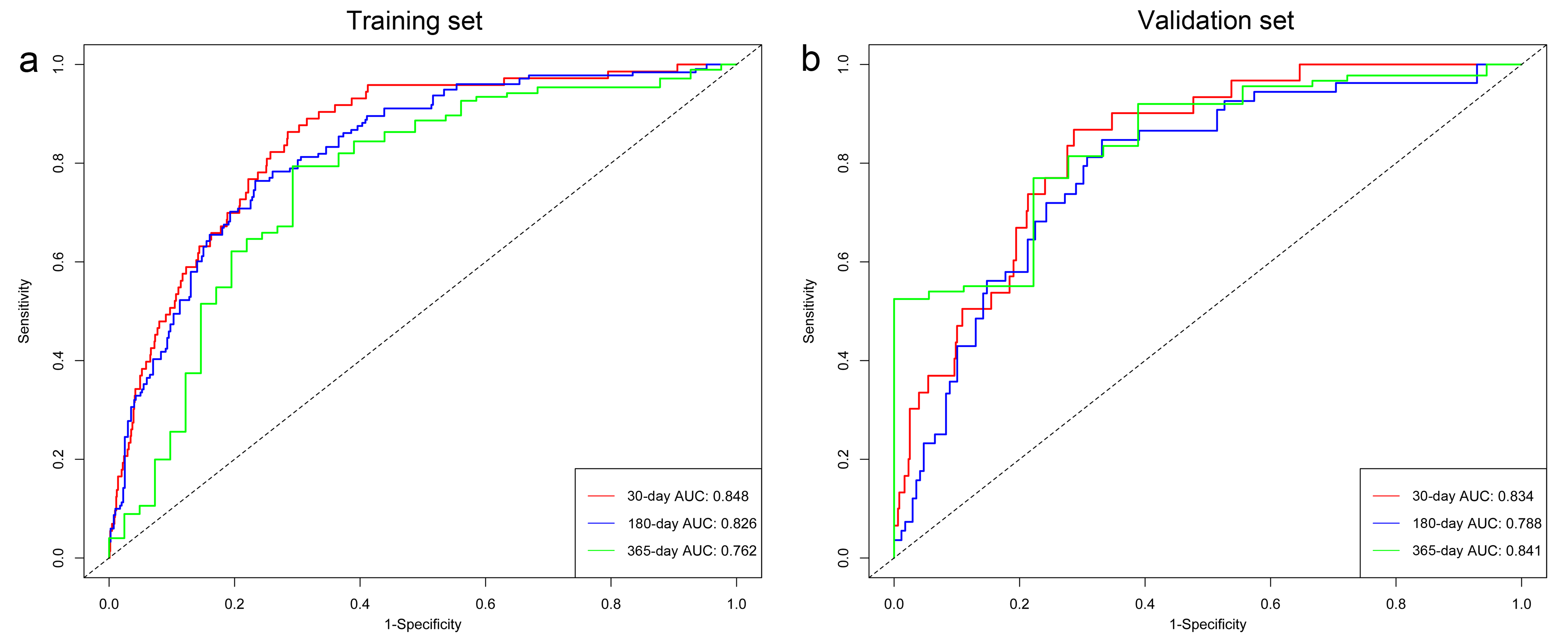

Supplement: Supplementary Figure 1 — Receiver operating characteristic (ROC) curves for 30-, 180-, and 365-day survival rates. (A) Training set. (B) Validation set. [file Image_1.TIF]

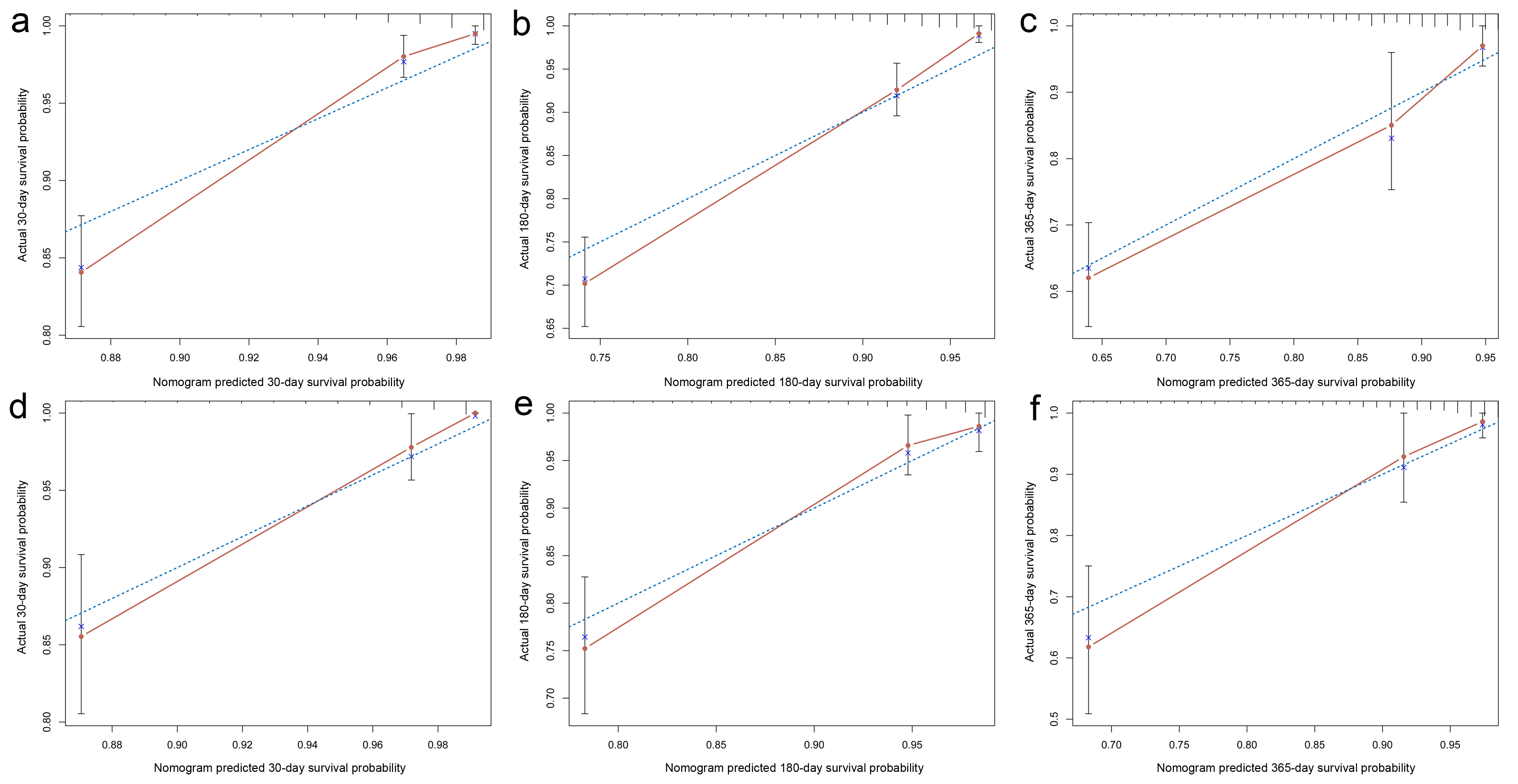

Supplement: Supplementary Figure 2 — Calibration curves for the nomogram in the training and validation sets. Y-axis represents the actual survival rate, while the X-axis represents the nomogram-predicted survival rate. The blue dotted line indicates perfect prediction by an ideal model. (A–C) 30-, 180-, and 365-day survival rates in the training set. (D–F) 30-, 180-, and 365-day survival rates in the validation set. [file Image_2.TIF]

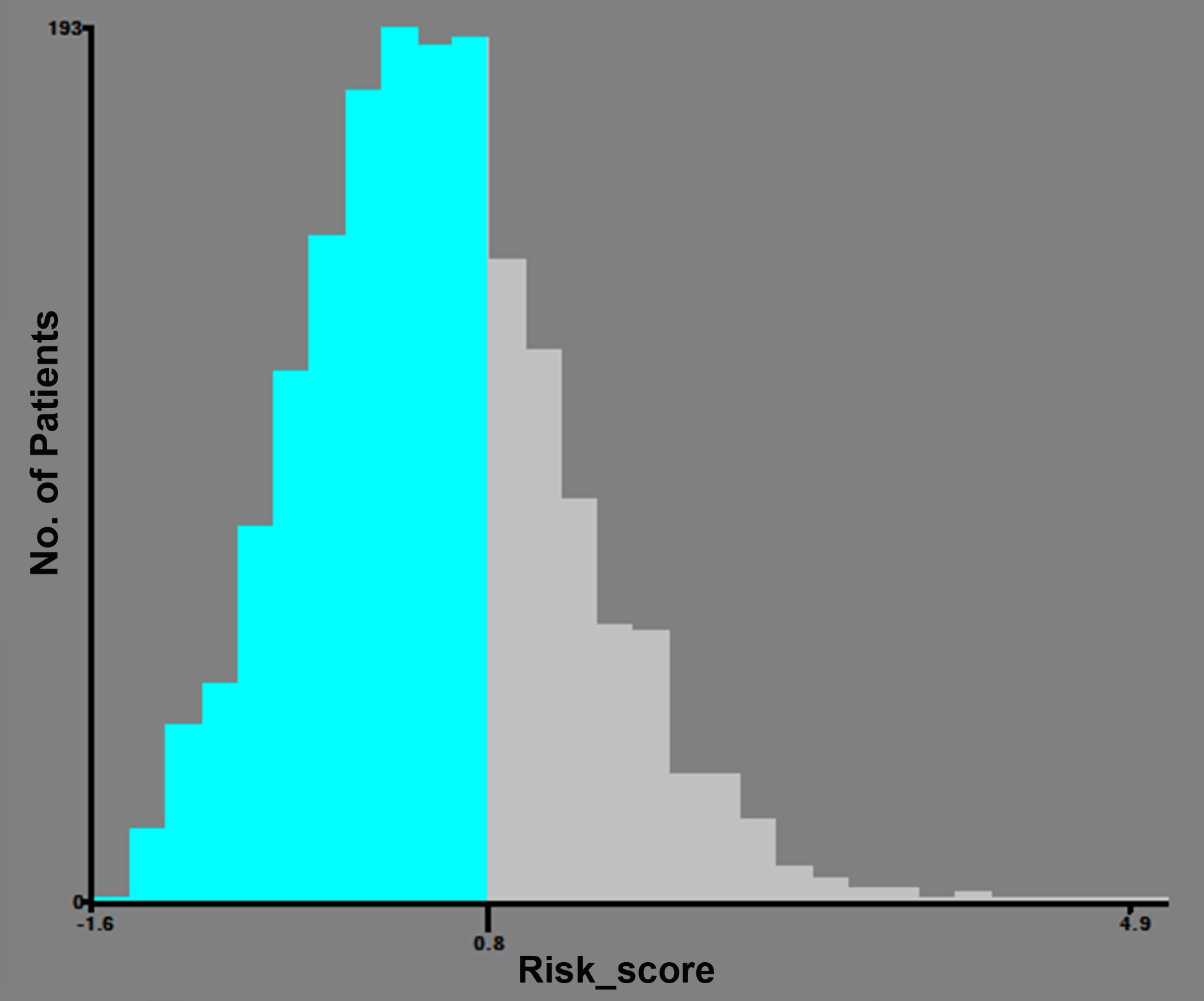

Supplement: Supplementary Figure 3 — Determination of the cut-off score, for the mortality risk stratification, using the X-tile program. A cut-off score ≤ 0.8 indicates low-risk, and > 0.8 indicates high-risk. [file Image_3.TIF]

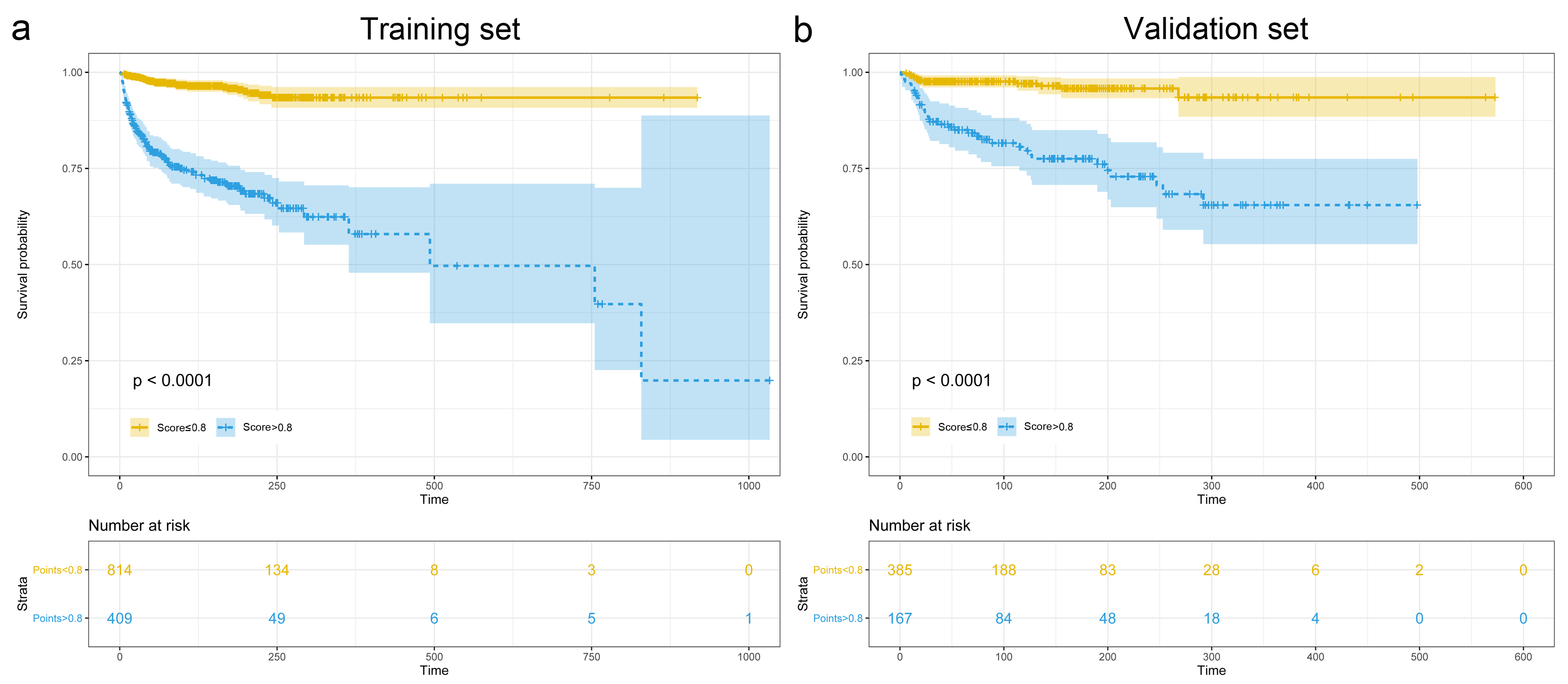

Supplement: Supplementary Figure 4 — Kaplan-Meier curves for the high-risk group and low-risk group in the training and validation sets. (A) Training set. (B) Validation set. [file Image_4.TIF]
