# Supplementary material for: Predicting All-Cause Mortality Risk in Atrial Fibrillation Patients: A Novel LASSO-Cox Model Generated From a Prospective Dataset
Source: Front Cardiovasc Med. 2021 Oct 18;8:730453. doi: 10.3389/fcvm.2021.730453 (PMC8558306; doi:10.3389/fcvm.2021.730453)
Supplement: Supplementary Table 1 — The 122 variables and the missing rates collected in the dataset. [file Table_1.docx]

**Supplemental Table 1. The 122 variables and the missing rates collected in the dataset**

| **Number** | **Variable** | **Missing rate** |
| --- | --- | --- |
| 1 | Age (years) | 0% |
| 2 | Gender: male | 0% |
| 3 | CHA_2_DS_2_-VASc score | 0% |
| 4 | HAS-BLED score | 0% |
| 5 | Diabetes mellitus | 0% |
| 6 | Atherosclerosis | 0% |
| 7 | Coronary stent | 0.16% |
| 8 | Pacemaker | 0.16% |
| 9 | Hypertension | 0% |
| 10 | Hysteromyoma | 0% |
| 11 | Stroke | 0% |
| 12 | Cerebral hemorrhage | 0% |
| 13 | Heart failure | 0.16% |
| 14 | Cardiac insufficiency | 0.16% |
| 15 | DCM | 0% |
| 16 | Rheumatic heart disease | 0.16% |
| 17 | Cancer | 0.16% |
| 18 | Renal insufficiency | 0.16% |
| 19 | COPD | 0.16% |
| 20 | Thyroid | 0.16% |
| 21 | Gastrointestinal bleeding | 0.16% |
| 22 | Hepatitis B virus | 0% |
| 23 | Operation | 0% |
| 24 | Current smoker status | 0% |
| 25 | Alcohol | 0% |
| 26 | Systolic blood pressure | 4.28% |
| 27 | Diastolic blood pressure | 4.28% |
| 28 | Anticoagulant treatment | 0% |
| 29 | Aspirin treatment | 0% |
| 30 | Clopidogrel treatment | 0% |
| 31 | Amiodarone treatment | 0% |
| 32 | β-Blocker treatment | 0% |
| 33 | Propranolol treatment | 0% |
| 34 | Digoxin treatment | 0% |
| 35 | Furosemide treatment | 0% |
| 36 | Torsemide treatment | 0% |
| 37 | Hydrochlorothiazide treatment | 0% |
| 38 | Angiotensin converting enzyme inhibitor treatment | 0% |
| 39 | Angiotensin receptor blockers treatment | 0% |
| 40 | Dihydropyridine treatment | 0% |
| 41 | Nondihydropyridine treatment | 0% |
| 42 | Statin treatment | 0% |
| 43 | Urobilinogen | 9.69% |
| 44 | Urine bilirubin | 9.69% |
| 45 | Urine ketone | 9.74% |
| 46 | Urinary occult blood | 9.74% |
| 47 | Urinary protein | 9.69% |
| 48 | Urine nitrite | 9.69% |
| 49 | Urinary glucose | 9.69% |
| 50 | Urine specific gravity | 9.69% |
| 51 | Urine pH | 9.69% |
| 52 | Urine vitamin C | 12.4% |
| 53 | Hemolysis | 5.35% |
| 54 | WBC | 8.90% |
| 55 | Neutrophil ratio | 8.90% |
| 56 | Lymphocyte ratio | 8.90% |
| 57 | Mononuclear cell ratio | 8.90% |
| 58 | Eosinophil ratio | 8.90% |
| 59 | Basophil ratio | 8.90% |
| 60 | Lymphocyte number | 8.90% |
| 61 | Mononuclear cell number | 8.90% |
| 62 | Eosinophil number | 8.90% |
| 63 | Basophil number | 8.90% |
| 64 | RBC | 8.90% |
| 65 | HGB | 8.73% |
| 66 | Hematocrit | 8.73% |
| 67 | MCV | 8.90% |
| 68 | MCH | 8.90% |
| 69 | MCHC | 8.90% |
| 70 | RDW-CV | 8.90% |
| 71 | RWD-SD | 8.90% |
| 72 | Platelet | 8.90% |
| 73 | MPV | 9.91% |
| 74 | PCT | 9.97% |
| 75 | PDW | 10.0% |
| 76 | Prothrombin time | 16.2% |
| 77 | Prothrombin time activity percentage | 16.3% |
| 78 | Prothrombin time ratio | 16.3% |
| 79 | PT-INR | 16.3% |
| 80 | Fibrinogen | 18.0% |
| 81 | K | 5.23% |
| 82 | Na | 5.23% |
| 83 | Cl | 5.23% |
| 84 | Ca | 5.23% |
| 85 | CO_2_ | 5.01% |
| 86 | GLU | 4.90% |
| 87 | BUN | 5.01% |
| 88 | Cr | 4.95% |
| 89 | CK | 10.0% |
| 90 | CKMB | 19.90% |
| 91 | Hydroxybutyrate dehydrogenase | 10.0% |
| 92 | Lactic dehydrogenase | 7.83% |
| 93 | Lactate dehydrogenase isoenzyme 1 | 14.5% |
| 94 | AST | 7.49% |
| 95 | ALT | 10.2% |
| 96 | Gamma-glutamyl transpeptidase | 11.4% |
| 97 | Alkaline phosphatase | 10.2% |
| 98 | CHE | 10.0% |
| 99 | MAO | 10.2% |
| 100 | Fucosidase | 10.2% |
| 101 | Total protein | 10.1% |
| 102 | Albumin | 10.1% |
| 103 | Globulin | 10.2% |
| 104 | Albumin globulin ratio | 10.2% |
| 105 | Total bilirubin | 10.0% |
| 106 | Direct bilirubin | 10.0% |
| 107 | Indirect bilirubin | 10.0% |
| 108 | Total cholesterol | 13.6% |
| 109 | Triglycerides | 13.6% |
| 110 | HDL | 13.6% |
| 111 | LDL | 13.6% |
| 112 | Left atrial dimension | 28.3% |
| 113 | LVD | 27.7% |
| 114 | Ejection fraction | 27.6% |
| 115 | IVS | 28.6% |
| 116 | LVPW | 28.6% |
| 117 | NE# | 8.90% |
| 118 | NLR | 8.90% |
| 119 | Embolism | 3.88% |
| 120 | Bleeding | 3.88% |
| 121 | Jaundice | 5.35% |
| 122 | Lipemia | 5.35% |

Abbreviations: DCM, Dilated cardiomyopathy; COPD, Chronic obstructive pulmonary disease; WBC, White blood cells; RBC, Red blood cells; HGB, Hemoglobin concentration; MCV, Mean corpuscular volume; MCH, Mean corpuscular hemoglobin; MCHC, mean corpuscular hemoglobin concentration; RDW-CV, red blood cell volume distribution width - coefficient of variation; RDW-SD, red blood cell volume distribution width - standard deviation; MPV, Mean platelet volume; PCT, Thrombocytopenia; PDW, Platelet distribution width; PT-INR, Prothrombin time international normalized ratio; GLU, Blood glucose; BUN, Blood urea nitrogen; Cr, Creatinine; CK, Creatine kinase; CKMB, Creatine kinase MB isoenzyme; AST, Aspartate aminotransferase; ALT, Alanine aminotransferase; MAO, Monoamine oxidase; CHE, Cholinesterase; HDL, High-density lipoprotein; LDL, Low-density lipoprotein; LVD, Left ventricular end-diastolic diameter; LVS, Left ventricular end- systolic diameter; LVPW, Left ventricular posterior wall thickness; NE#, Neutrophil number; NLR, Neutrophil-lymphocyte ratio.
