# Supplementary material for: Predicting All-Cause Mortality Risk in Atrial Fibrillation Patients: A Novel LASSO-Cox Model Generated From a Prospective Dataset
Source: Front Cardiovasc Med. 2021 Oct 18;8:730453. doi: 10.3389/fcvm.2021.730453 (PMC8558306; doi:10.3389/fcvm.2021.730453)
Supplement: Supplementary Table 2 — Top-20 variables selected by the RF algorithm. [file Table_2.docx]

**Supplemental Table 2. Top-20 variables selected by the RF algorithm**

| **Number** | **Variable** |
| --- | --- |
| 1 | MAO |
| 2 | NLR |
| 3 | LYN |
| 4 | LY |
| 5 | CHE |
| 6 | BUN |
| 7 | NE |
| 8 | Cl |
| 9 | GLU |
| 10 | NE# |
| 11 | Cr |
| 12 | Age |
| 13 | MCV |
| 14 | HDL |
| 15 | MCH |
| 16 | BA |
| 17 | CKMB |
| 18 | WBC |
| 19 | Ca |
| 20 | CK |

Abbreviations: MAO, Monoamine oxidase; NLR, Neutrophils/lymphocytes; LYN, Lymphocyte number; LY, Lymphocyte ratio; CHE, Cholinesterase; BUN, Blood urea nitrogen; NE, Neutrophil ratio; Cl, Chlorine; GLU, Blood glucose; NE#, Neutrophil number; Cr, Creatinine; MCV, Mean corpuscular volume; HDL, High-density lipoprotein; MCH, Mean corpuscular hemoglobin; BA, Basophil ratio; CKMB, Creatine kinase MB isoenzyme; WBC, White blood cells; CK, Creatine kinase.
